# Vertebral bone microarchitecture and osteocyte characteristics of three toothed whale species with varying diving behaviour

**DOI:** 10.1038/s41598-017-01926-7

**Published:** 2017-05-09

**Authors:** Tim Rolvien, Michael Hahn, Ursula Siebert, Klaus Püschel, Hans-Joachim Wilke, Björn Busse, Michael Amling, Ralf Oheim

**Affiliations:** 1 Department of Osteology and Biomechanics, University Medical Center Hamburg-Eppendorf, Lottestr. 59, 22529 Hamburg, Germany; 2 Institute for Terrestrial and Aquatic Wildlife Research, University of Veterinary Medicine Hannover, Foundation, Werftstrasse 6, 25761 Buesum, Germany; 3 Department of Forensic Medicine, University Medical Center Hamburg-Eppendorf, Butenfeld 34, 22529 Hamburg, Germany; 4 Institute of Orthopedic Research and Biomechanics, University Medical Center Ulm, Helmholtzstraße 14 D, 89081 Ulm, Germany

## Abstract

Although vertebral bone microarchitecture has been studied in various tetrapods, limited quantitative data are available on the structural and compositional changes of vertebrae in marine mammals. Whales exhibit exceptional swimming and diving behaviour, and they may not be immune to diving-associated bone pathologies. Lumbar vertebral bodies were analysed in three toothed whale species: the sperm whale (*Physeter macrocephalus)*, orca (*Orcinus orca)* and harbour porpoise (*Phocoena phocoena)*. The bone volume fraction (BV/TV) did not scale with body size, although the trabeculae were thicker, fewer in number and further apart in larger whale species than in the other two species. These parameters had a negative allometric scaling relationship with body length. In sperm whales and orcas, the analyses revealed a central ossification zone (“bone-within-bone”) with an increased BV/TV and trabecular thickness. Furthermore, a large number of empty osteocyte lacunae was observed in the sperm whales. Quantitative backscattered electron imaging showed that the lacunae were significantly smaller and less densely packed. Our results indicate that whales have a unique vertebral bone morphology with an inside-out appearance and that deep diving may result in a small number of viable osteocytes because of diving depth-related osteocyte death.

## Introduction

Animals living in marine environments require a skeleton that is specifically optimised for swimming and diving in conditions of changed gravity, density and dysbarism. However, this adaptation may not always be successfully implemented. For example, deep diving causes a dysbaric osteonecrosis from end-artery nitrogen embolism (“the bends”) in sperm whales and beaked whales, and these animals may not be immune to deep diving-related pathologic bone conditions that were initially described in diving humans^1–4^. Sperm whales are the second deepest diving mammal and can reach depths of over 2000 metres^5^. However, the diagnosis of osteonecrosis has not been histologically confirmed and has been questioned^6^. Moreover, whale vertebral bone microarchitecture has not been previously analysed histologically; thus, their bone biology remains unclear.

In addition to osteonecrosis, pathological bone conditions, such as degenerative changes, osteomyelitis or spondyloarthropathy, have been observed in whales^1, 7^. The forehead of sperm whales contains two large oil-filled compartments, the “spermaceti organ”^8^ and “junk”, which decrease the skull stress of sperm whales and may be specialised for ramming combat^9^. An energy absorptive, flexible skull amphitheatre occurs at the dorsal side of the skull, and it may prevent fractures of possibly weakened bone in the vertebral column^10^.

Vertebral bodies in mammals are typically composed of an outer cortical and inner trabecular bone compartment^11^. Structurally, the bone matrix is composed of immature woven bone and lamellar bone that form osteons. Within the bone matrix, mechanosensitive bone cells called osteocytes are entrapped and represent the most abundant cell type in the skeleton. These cells are connected via nanometre-sized channels called canaliculi and act as the main regulator of bone remodelling through their ability to guide bone-forming osteoblasts and bone-resorbing osteoclasts on bone surfaces^12, 13^. Osteocyte death can occur under various conditions, such as ageing or osteonecrosis^14, 15^, and osteocyte deficiency represents metabolically inactive dead bone and may occur in deep-diving whales. Notably, bone completely devoid of osteocytes (i.e., anosteocytic) is found in many fish species (i.e., advanced teleost fish, “acellular bone”)^16, 17^.

Whether trabecular thickness scales with animal size in different whale species is currently unknown. Moreover, the differences in vertebral microanatomy between whales and other terrestrial and aquatic mammals are poorly understood, particularly considering the varying diving-depth behaviours of whale species. In this study, the vertebral bodies of three toothed whale species (odontocete cetaceans) were comparatively studied to gain deeper insights into mammalian bone biology in marine environments. Fresh bone specimens were collected from whale cadavers as twelve sperm whales (*Physeter macrocephalus)* stranded on the beaches of northern Germany in early 2016. The cause of the sperm whale stranding event around the North Sea remains unclear; however, weather changes may be the most likely factor that misdirected whales into shallow waters. In the same period, an orca (*Orcinus orca)* and several harbour porpoises (*Phocoena phocoena)* were also found stranded on the coast of the North Sea.

## Results

### Whale characteristics

Age was determined via annual growth layer group (GLG) analyses of the teeth of sperm whales, and the results revealed that the three sperm whales in this study were 12, 13 and 15 years of age and had body lengths of 10.5, 10.95 and 11.4 metres, respectively. The results of a necropsy indicated that the animals were healthy and died of cardiovascular failure caused by stranding. The orca measured 2.5 m, and the harbour porpoise was 1.05 m, indicating that neither of these animals were fully mature.

### Bone microstructure

**Figure 1:**
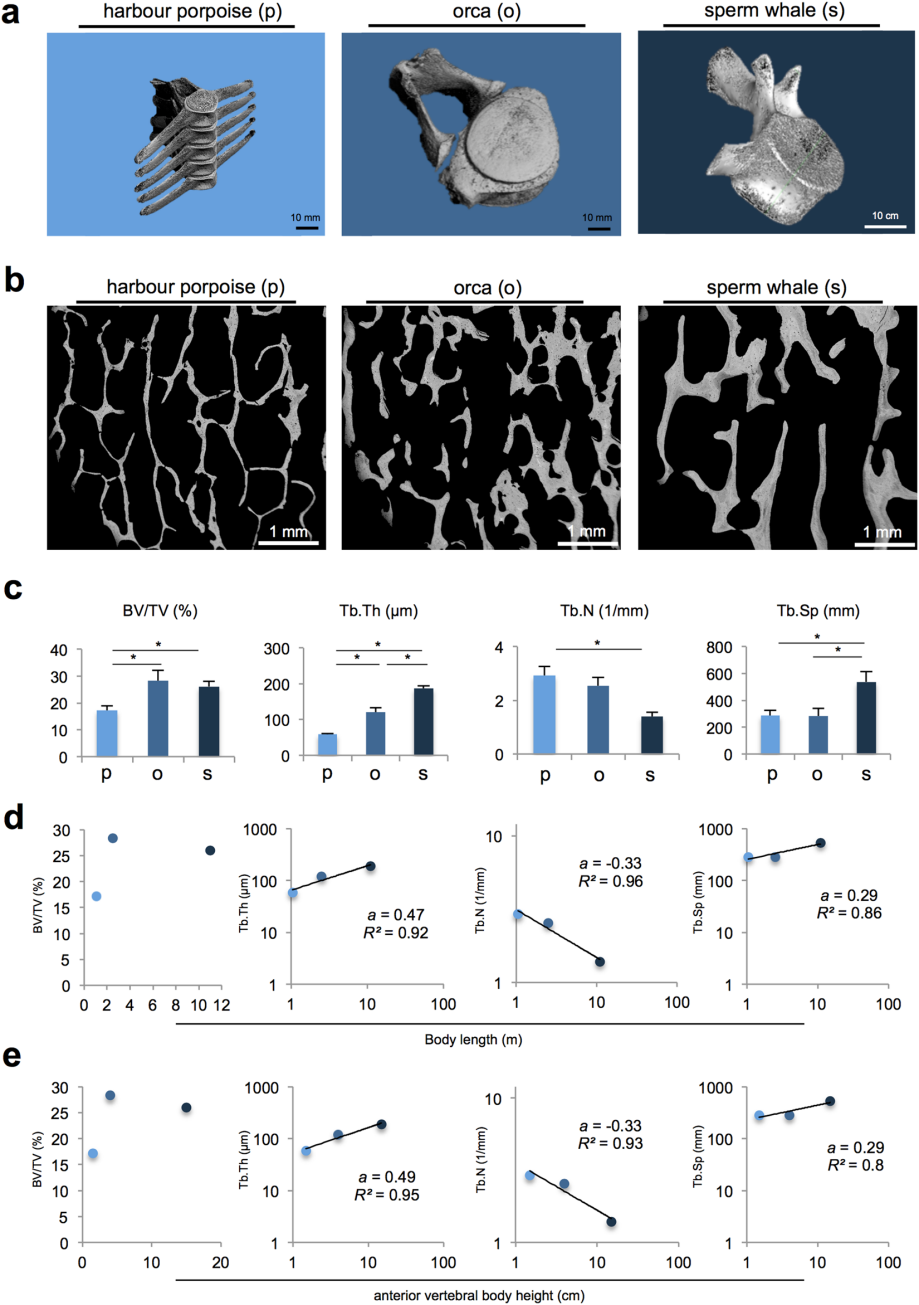
Bone morphometry and scaling relationship of L1. (**a**) Three-dimensional reconstruction of the lumbar vertebral body L1 of the harbour porpoise (p), orca (o) and sperm whale (s). (**b**) Overview of the bone microstructure of the three whale species. Images obtained by backscattered electron microscopy (BSEM). (**c**) Comparative histomorphometric analyses revealed significant differences between the three whale species, with the sperm whales showing the thickest, fewest and most separated trabeculae and the orca showing the highest bone volume fraction. *P < 0.05. (**d**) Trabeculae scale with body size and show a negative allometric relationship for Tb.Th, Tb.N and Tb.Sp but not BV/TV. (**e**) Anterior vertebral body height and bone structure parameters present the same negative allometric scaling relationships.

All three whale species showed a mammalian-like morphology of the lumbar vertebral bodies, with transverse and spinous processes based on computed tomography (CT) and high-resolution quantitative computed tomography (HR-pQCT) imaging (Fig. 1a, for size and weight see Table 1). Subsequent comparative histomorphometric analyses of the lumbar vertebral bodies revealed differences in the bone microstructures among the three whales. Notably, a definite transition between the trabecular and cortical bone compartment was not observed, and the cortical bone was very thin. Representative images of the trabecular microstructures assessed via backscattered electron microscopy (BSEM) are shown in Fig. 1b.

**Table 1:** Size and weight of the vertebral body L1 and diving depth^5, 30, 31^ for each whale species.

	L1 size (cm)	L1 weight (kg)	Diving depth (m)
Harbour porpoise	1.5 × 4.8 × 6.5	0.01	−200
Orca	4 × 13 × 18.5	0.27	−200
Sperm whale	15 × 50 × 70	7.5	−2000

The mean bone volume fraction (BV/TV) was highest in the orca (28.3%) compared with the harbour porpoise (17.2%) and sperm whales (26%). The absolute trabecular thickness was the highest in the sperm whales, followed by the orca and harbour porpoise (Fig. 1c). The trabecular number decreased and the trabecular separation increased between the harbour porpoise, orca and sperm whale (Fig. 1c).

Of the three different-sized whale species, the BV/TV did not scale with body size (no linear or exponential correlation; Fig. 1d). The trabecular thickness, separation and number changed to a lesser extent compared with the body length, indicating a negative allometric relationship for all three parameters (Fig. 1d). The bone structural parameters were also correlated with the anterior vertebral body height of L1, which confirmed a negative trabecular allometry for trabecular thickness, number and separation (Fig. 1e).

### “Bone-inside-bone” appearance of vertebral bodies

**Figure 2:**
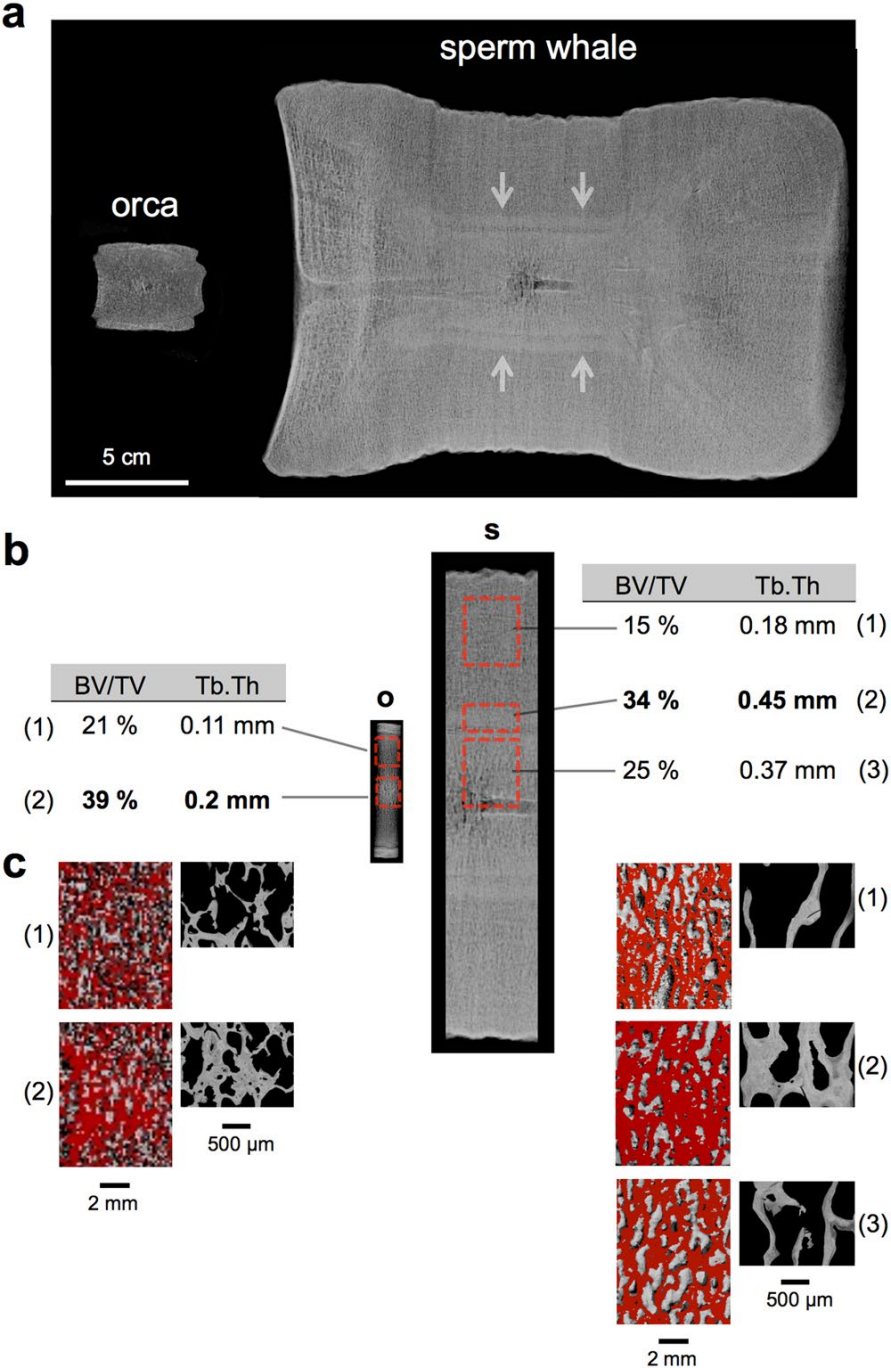
Central bone densification (bone-within-bone) in orca and sperm whale. (**a**) Orcas and sperm whales show a central ossification zone with contact radiography (midsagittal slice of 0.8 cm). Note the dimensions of the vertebral bodies. (**b**) Punch biopsies from endplate to endplate were taken. In the orca (o) and sperm whales (s), further quantification of the different regions of interest (red boxes) using HR-pQCT revealed an increased bone volume fraction (BV/TV) with thicker trabeculae (Tb.Th) in the central parts, and it corresponded to the “bone-within-bone” structure seen in radiographies. (**c**) Representative images of the scanned subregions in HR-pQCT indicate that the trabecular network was denser in the central parts.

In sperm whales as well as orca, a central ossification zone radiologically described as “bone-inside-bone” was observed in radiographic studies (Fig. 2a). Sagittal and coronal CT reconstructions confirmed this box-shaped densification inside the vertebral bodies. The “bone-inside-bone” appearance and regional heterogeneity of the lumbar vertebral bodies was further quantified by HR-pQCT. In the orca and sperm whales, the BV/TV and trabecular thickness within the ossification zone were considerably higher than that in the peripheral parts (Fig. 2b,c).

**Figure 3:**
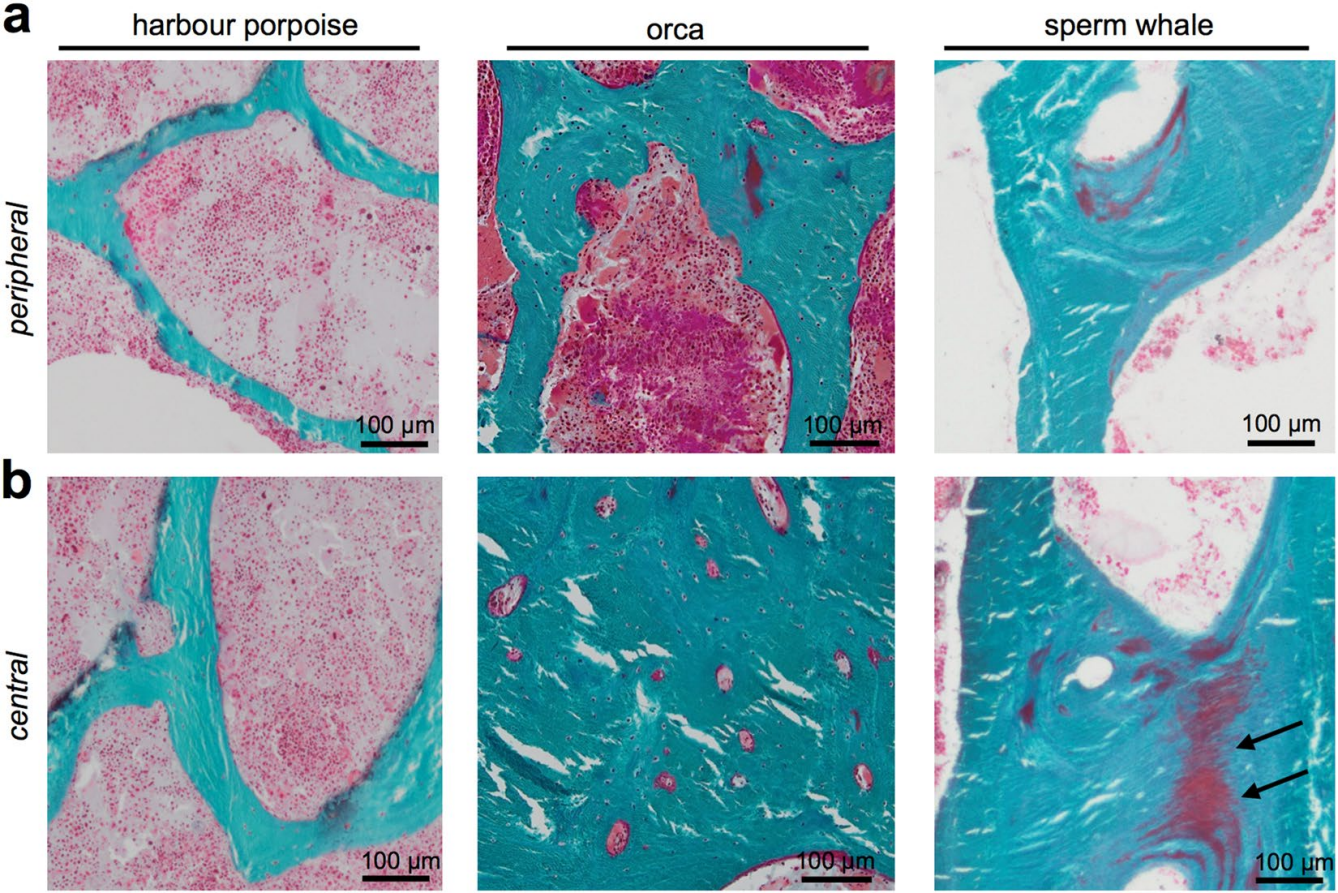
Histology of the vertebral bodies. (**a**) In peripheral zones, the trabeculae were thinner and more separated. (**b**) In the centre of the vertebral body of orcas and sperm whales, the trabeculae converged to a dense structure with a compact appearance that included osteonal structures (Masson Goldner staining, 50x magnification). Harbour porpoise vertebral bone did not show this phenomenon.

The HR-pQCT results were confirmed via histologic measurements. In orcas and sperm whales, the trabeculae converged to a dense structure in the central parts of the vertebral bodies. Here, osteonal entities (i.e., secondary osteons) were observed in the centre of trabeculae (Fig. 3a,b). Furthermore, areas of non-mineralised bone matrix within the bone, which are called buried osteoid, were identified in the sperm whales (Fig. 3b, black arrows). Of note, scant bone marrow with few blood cells was observed in the marrow space of the sperm whales (Fig. 3a,b right column). The harbour porpoise did not show central bone densification.

### Bone mineral density distribution analysis (BMDD)

**Figure 4:**
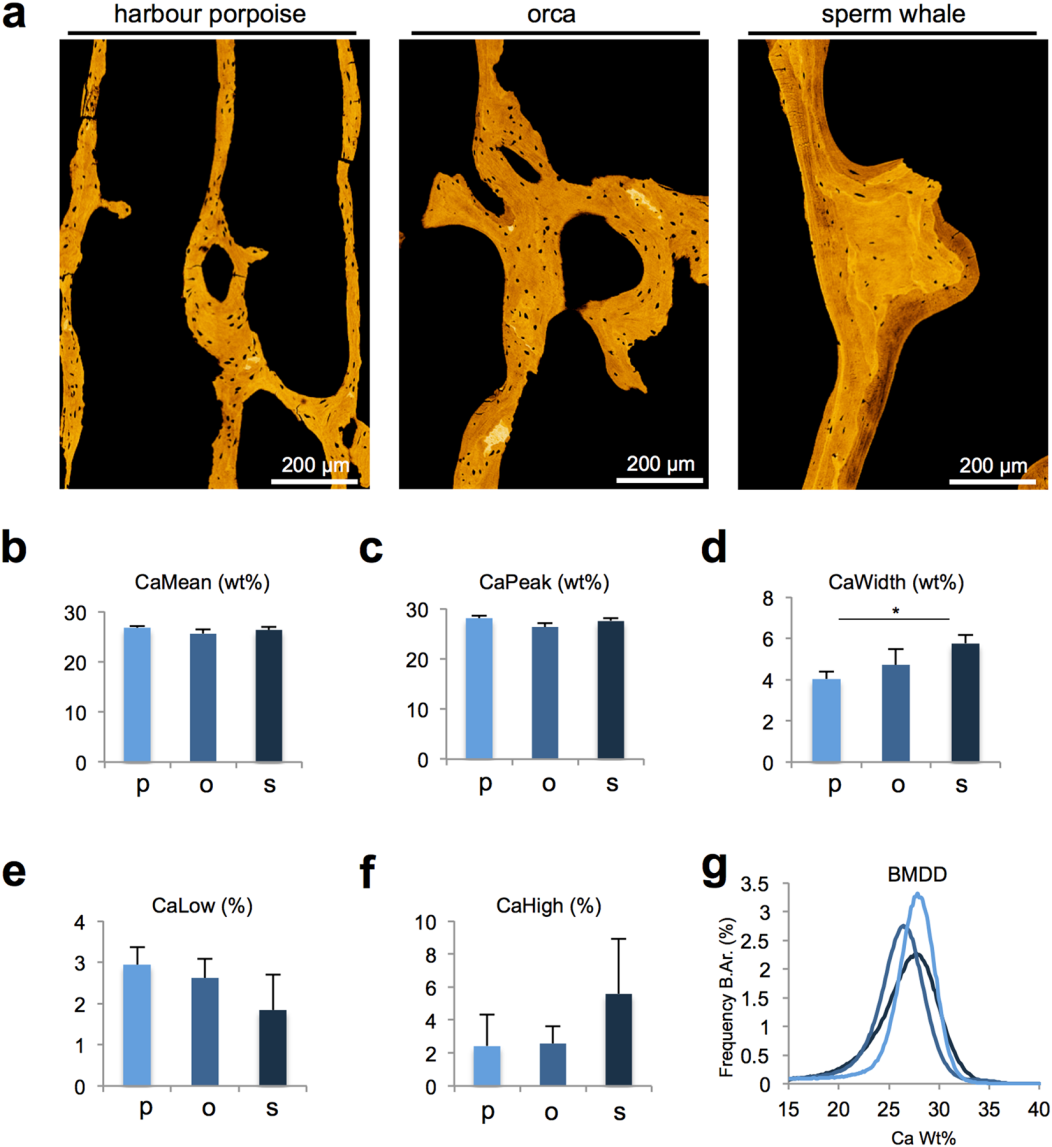
Quantitative backscattered electron imaging (qBEI) of the trabecular bone. (**a**) Pseudo-coloured images of the trabecular bone. (**b**–**f**) CaMean, CaPeak, CaLow and CaHigh did not differ between the groups, indicating a similar mineralisation pattern, whereas CaWidth was increased significantly in sperm whales, which indicates higher mineralisation heterogeneity. (**g**) Histogram of the bone mineral density distribution (BMDD).

Quantitative backscattered electron imaging (qBEI) of the vertebral trabecular bone compartment revealed an overall trabecular bone mineral density distribution (BMDD) that did not differ among the three whale species (Fig. 4a–g). However, the sperm whale showed a significantly higher heterogeneity of mineralisation (CaWidth), indicating both high and low mineralised areas and more heterogeneous bone matrix mineralisation (Fig. 4d).

### Osteocyte deficiency in sperm whales

**Figure 5:**
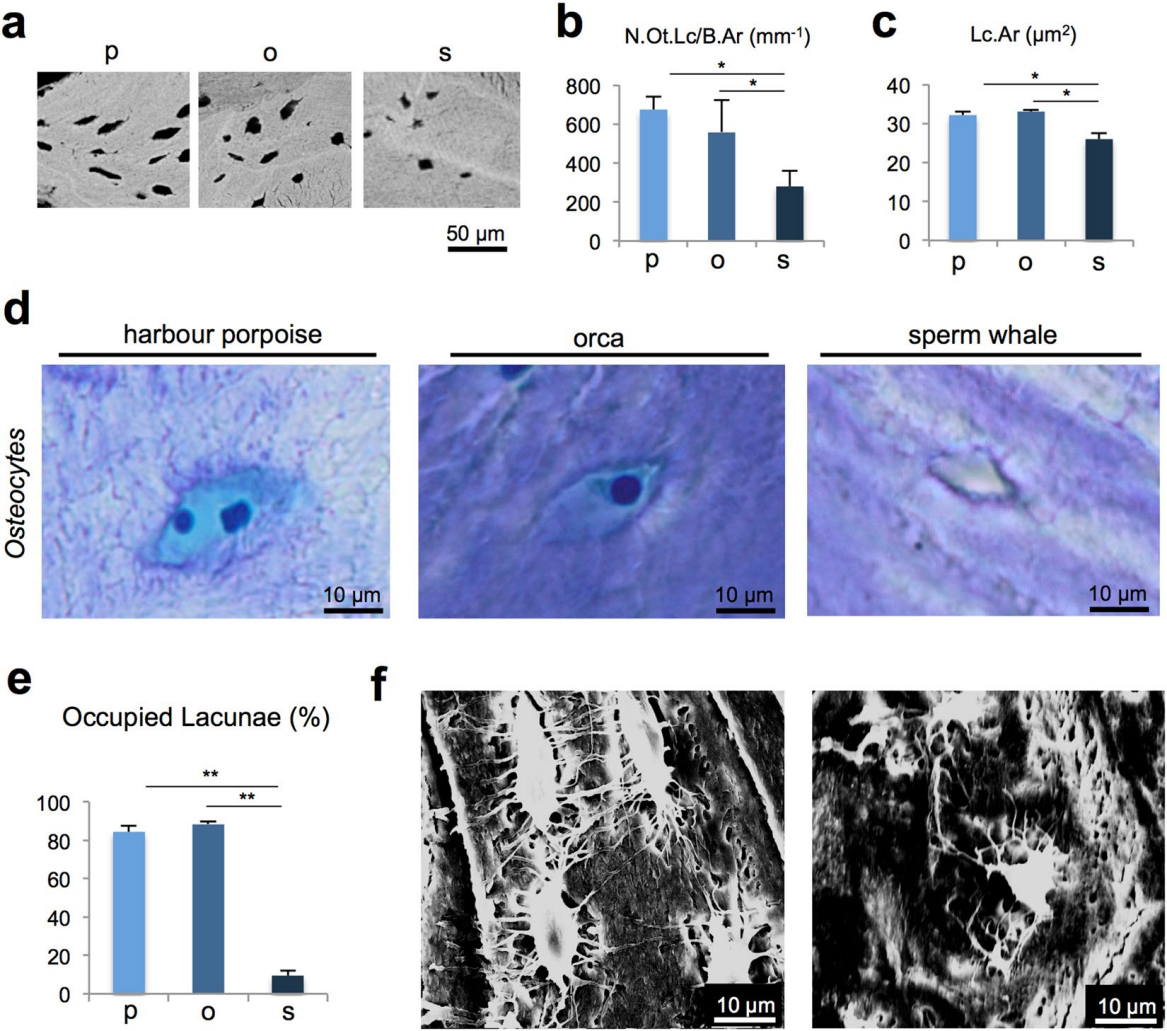
Osteocyte characteristics. (**a**) Representative images obtained using BSEM show the differences in lacunar number and size. (**b**) The number of osteocyte lacunae per bone area (N.Ot.Lc/B.Ar) was significantly lower in the sperm whales than that in the other whale species. (**c**) Osteocyte lacunar area (Lc.Ar) was smaller in the sperm whales. *P < 0.05. (**d**) Toluidine blue-stained sections of undecalcified bone specimens revealed that the harbour porpoise had several osteocytes with 2 cell nuclei, the orca had typically shaped osteocytes and the sperm whale showed only small and empty lacunae; 400x magnification. (**e**) Percentage of occupied (viable) lacunae was significantly decreased in the sperm whales. **P < 0.001. (**f**) Images of the osteocyte lacunar connections performed via acid etching of the acrylate embedded specimens followed by scanning electron microscopy (SEM); the images show a well-developed lacunocanalicular system in the orca (left image), whereas poor connections via canaliculi are observed among osteocytes in the sperm whale bone (right image).

Osteocyte lacunar analyses showed that the sperm whales had significantly fewer osteocyte lacunae (N.Ot.Lc) and smaller osteocyte lacunae (Lc.Ar) than the harbour porpoises and orcas (Fig. 5a–c). Histologic quantification of the osteocytes revealed a significantly lower percentage of occupied lacunae in the sperm whales compared with the other two whale species, indicating a high percentage of empty lacunae (i.e., identification of dead osteocytes) (Fig. 5d,e). The harbour porpoise had several osteocyte lacunae with 2 cell nuclei (Fig. 5d). Scanning electron microscopy (SEM) analyses of acid-etched bone specimens demonstrated that compared with the orca, the sperm whales had a poorly connected canalicular network (Fig. 5f).

## Discussion

This study provides new insights into the structure, composition and function of vertebral bone tissues of toothed whale species in marine environments. Bone histomorphometric analyses revealed that the trabecular composition of the vertebral bodies is similar to that of other mammalian species and presents typical fibrolamellar bone and a unique and size-dependent trabecular organisation^11, 18^. In general, two major functional adaptations have been identified in vertebrates adapted to aquatic life, depending on the functional requirements: increased bone mass (i.e., osteosclerosis) and pronounced spongious organisation^19^. Previous studies on extinct mosasauroids have shown that adaptations to aquatic life are not always consistent with the overall bone mass increase or decrease and may be more closely associated with a peculiar trabecular microarchitecture that varies depending on the size of the species^20^. For vertebral microanatomy, aquatic taxa have a higher number of relatively thin trabeculae^18^. Comparative studies on the inner vertebral bone architecture in terrestrial and aquatic mammals have established locomotion categories for trabecular bone parameters, and the most evident changes were observed with increased body size^11^.

Our results are consistent with previous observations in other taxa showing that the BV/TV may not be correlated with animal size. The trabeculae in larger whale species are thicker, further apart and fewer per unit volume than those in smaller animals, indicating a trade-off between trabecular number and thickness^21^. In fact, we observed a negative allometric scaling relationship between body size and trabecular thickness, trabecular number number and trabecular separation, which indicates that these changes are less pronounced than the increase in whale body length; thus, the animals are able to withstand higher loads without a commensurate increase in bone mass^22^.

Importantly, our results indicate that biomechanical adaptations to swimming may have occurred, thereby forming a “bone-inside-bone” structure with higher bone mass in the centre of the vertebral body. Cortical bone was nearly absent, whereas the trabecular bone became more compact in the centre with the existing intra-trabecular osteon-like structures (Figs 2 and 3a). In humans, “bone-inside-bone” conditions have been described only in disease states, such as osteopetrosis, which is caused by an osteoclastic resorption defect^23^, whereas in most bony fish, such as zebrafish, bone mass accumulation in the centre of the vertebral body similar to an “inside-out” bone distribution pattern is also found in the vertebral bodies (Supplemental Figure)^24, 25^. In lordotic fish vertebrae, the highest strain regions are in the vertebral centrum^26^.

In whales, regional variations in bone density have been observed in previous studies on humpback whales and at other skeletal sites, such as the mandible, where the bone geometry and density distribution are adapted to bending forces^27, 28^. In the spine, biomechanical simulation models are needed to further characterise the vertebral bone adaptations to swimming activity. Such investigations should involve numerical techniques, including the finite element method (FEM), and they will require image data sets with high resolution and should include complete vertebral bodies for each species.

The accumulation of buried non-mineralised bone matrix (buried osteoid) found in the histologic analysis of the sperm whales suggests mineralisation defects^29^. The prevalent buried osteoid was consistent with increases in mineralization heterogeneity (CaWidth) as detected by qBEI and may have been caused a decelerated bone remodelling process in sperm whales compared with the other two whale species (i.e., no viable osteocytes and no osteoblasts and/or osteoclasts attached to the bone surface).

The detected osteocyte deficiency in sperm whales may occur for several reasons. First, in the marine environment, the organism is not subject to the same gravitational forces observed in terrestrial environments; thus, osteocytic pathways may not be needed. Second, because deep diving causes osteonecrosis, osteocyte deficiencies in sperm whales may be caused by maladaptations to deep diving^1^. Nitrogen embolisms could proceed through blood vessels into bone and affect the dendrites in the osteocyte canaliculi. Our results support this theory because orcas and harbour porpoises do not dive as deeply as sperm whales^30, 31^ (see Table 1), and their bones feature comparatively higher numbers of viable osteocytes in larger osteocyte lacunae. Similar to the bone turnover rate^32^, the osteocyte density of the trabecular bone may decrease with the size of the species, which is also consistent with our findings. Finally, phylogenetic drift towards anosteocytic bone was previously observed in many fish species and may represent a development in aquatic mammals, such as whales; however, whales cannot be physiologically compared with fish^33, 34^.

An enlargement of osteocyte lacunae, called osteocytic osteolysis, was not observed in our analyses. It has been reported in calcium-deprived conditions, such as vitamin D deficiency or lactation^35, 36^. Cetaceans regulate their water metabolism via the uptake of liquids from food, seawater and their body fat^37, 38^. Therefore, electrolyte regulation is fully adapted to the ingestion of salt water, which is calcium rich, and imbalanced calcium levels likely do not occur in whales.

Taken together, the lower osteocyte lacunar number, smaller osteocyte lacunar size, low osteocyte canalicular network connectivity, reduced osteocyte viability and limited vital bone marrow supply indicate that sperm whales possess more metabolically inactive bones (i.e., “dying” bones) compared with other whale species. These changes may be attributed to differences in diving behaviours among whale species. A larger central bone volume in sperm whale and orca vertebrae represents a unique feature indicative of a biomechanical adaptation to swimming. Nevertheless, the basic trabecular bone structure in the vertebral bodies of the three examined whale species is equal to that of humans or other mammals and presents a negative allometric scaling relationship with body size.

## Methods

### Specimens

The vertebral body L1 was collected from three sperm whales stranded on the coast of Schleswig-Holstein, Germany. For comparison, the L1 from the orca and harbour porpoises was extracted. All bone samples were collected within 7 days of death. The studied species are globally protected under Appendix I and II of CITES (Convention on International Trade in Endangered Species). Therefore, the specimens were transferred with special documentation of no commercial or trading interests according to the environmental authority of Schleswig-Holstein. We confirm that the experimental protocols were approved by the Institute for Terrestrial and Aquatic Wildlife Research (ITAW) on behalf of the Schleswig-Holstein Wadden Sea National Park.

### Age determination

Age determination was performed using the number of annual growth layer groups (GLGs) in the teeth of sperm whales^39^. Teeth were cut in half from root to crown, polished, and then immersed with the polished side face down in a 10% formic acid solution for 30 hours. Subsequently, the teeth were rinsed with running water and left to naturally dry at room temperature. The cutting surface was coloured with a soft pencil to highlight the structure. GLGs were counted under a magnifying glass.

### Radiography, computed tomography (CT) and high-resolution peripheral quantitative computed tomography (HR-pQCT)

The complete vertebral bodies of the sperm whales were scanned via CT (Philips Brilliance 16, Philips, Amsterdam, The Netherlands) with a layer thickness of 0.8 mm. The L1 of the orca and harbour porpoise as well as the subdivided parts of the sperm whale’s L1 were scanned via HR-pQCT (Xtreme-CT®, Scanco Medical, Bruettisellen, Switzerland) as previously described^40^. A tissue volume of 1000 mm^3^ for the orca and 8000 mm^3^ for the sperm whale was scanned for each region of interest. Mid-sagittal slices with a thickness of 0.8 cm were cut from the lumbar vertebral bodies and then scanned using a digital radiography device (Faxitron X-ray, Illinois, USA).

### Bone preparation and histology

Because of their size, the sperm and orca vertebral bodies were subdivided into peripheral (superior, inferior) and central parts, fixed in 3.7% PBS-buffered formaldehyde for seven days, dehydrated in ascending concentrations of ethanol and embedded in methacrylate. The 5-μm-thick sections were cut using a Microtec rotation microtome (Techno-Med; Munich, Germany). The sections were stained according to standard protocols of von Kossa/van Gieson, trichrome Goldner, and toluidine blue.

### Histomorphometry

The histomorphometric analyses were performed on non-decalcified toluidine blue and trichrome Goldner sections of the vertebral biopsies. Analyses of the BV/TV (%), trabecular thickness (Tb.Th, µm), trabecular number (Tb.N, 1/mm) and trabecular separation (Tb.Sp, mm) were performed according to ASBMR standards using the OsteoMeasure histomorphometry system (Osteometrics; Atlanta, GA, USA) connected to a Zeiss microscope (Carl Zeiss; Jena, Germany)^41^. The percentage of occupied lacunae was defined as the number of osteocytes with visible cell nuclei divided by the total number of osteocyte lacunae.

### Quantitative backscattered electron microscopy

Quantitative backscattered electron imaging (qBEI) was performed to assess the bone mineral density distribution (BMDD) as previously described^42–44^. Embedded specimens were polished and carbon coated for qBEI analyses. The scanning electron microscope (LEO 435 VP, LEO Electron Microscopy Ltd., Cambridge, England) was operated at 20 kV and 680 pA at a constant working distance (BSE Detector, Type 202, K.E. Developments Ltd., Cambridge, England). The grey values of the backscattered signal intensities correlate with the calcium content (weight-%) of the cross-sectioned vertebral cancellous bone^45^. The following parameters were evaluated: mean calcium concentration (CaMean, wt%), most frequent calcium concentration (CaPeak, wt%), standard deviation of the calcium distribution (CaWidth, wt%), percentage of bone area that is mineralized below the 5th percentile (CaLow, % bone area) and percentage of bone area containing Ca concentration above the 95th percentile (CaHigh, % bone area). The acquired images were thresholded using ImageJ software (ImageJ, 1.49 v, National Institutes of Health, USA–imagej.nih.gov/ij/) and the osteocyte number (N.Ot/B.Ar, 1/mm^2^) and lacunar area (Lc.Ar, µm^2^) were calculated from the images (200x magnification).

### Acid etching

The embedded bone samples from the qBEI analysis were re-polished and exposed to acid etching. This procedure was performed following the recommendations of Kubek *et al*.^46^ and repeated to visualise the lacuno-canalicular network^13^. The specimens were immersed with the polished side up in 9% phosphoric acid for 20 seconds followed by a short rinse in deionised water (1–2 seconds) and exposure in 5% sodium hypochlorite for 5 minutes with a final rinse in deionised water.

### Statistical analysis

For the vertebral bodies of each species, histomorphometric analyses were performed on three slices for a standardised region of interest relative to the size of the sectioned bone, and each vertebral body was scanned from the surface to the centre. The results are presented as the mean ± standard deviation. Inter-group differences were calculated via an ANOVA. P-values < 0.05 were considered statistically significant. The allometric scaling relationships were tested with a double-logarithmic plot and subsequent linear regression analysis. The regression coefficients (*R* ^*2*^) and allometry coefficients (*a*) were determined. For the regional comparison, absolute values of the subregions of interest are presented.

## Electronic supplementary material

Supplemental Figure
