# Supplementary material for: Vertebral bone microarchitecture and osteocyte characteristics of three toothed whale species with varying diving behaviour
Source: Sci Rep. 2017 May 9;7:1604. doi: 10.1038/s41598-017-01926-7 (PMC5431672; doi:10.1038/s41598-017-01926-7)
Supplement: Supplementary file 1 — Supplemental Figure [file 41598_2017_1926_MOESM1_ESM.pdf]

Supplemental Figure:

“Vertebral bone microarchitecture and osteocyte characteristics of three toothed whale species with varying diving behaviour.” by T Rolvien, M Hahn, U Siebert, K Püschel, H-J Wilke, B Busse, M Amling, and R Oheim

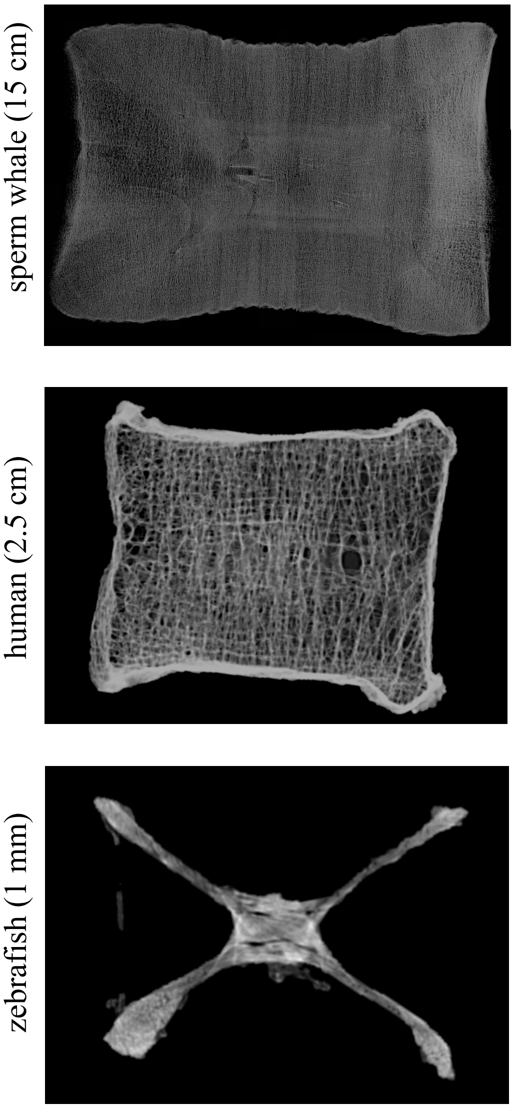

Contact radiography of the L1 vertebral body indicates the differences among sperm whale, human and zebrafish. While human vertebral bodies are composed of cortical bone and a relatively homogenous trabecular structure, both whales and zebrafish (as well as most other fish) show a central concentration of bone mass (Anterior vertebral body height in parentheses).
